# Risk of unsafe abortion associated with long-term contraception behaviour: a case control study from Sri Lanka

**DOI:** 10.1186/s12884-017-1376-7

**Published:** 2017-06-29

**Authors:** Carukshi Arambepola, Lalini C Rajapaksa

**Affiliations:** 1 Department of Community Medicine, Faculty of Medicine, University of Colombo, Kynsey Road, Colombo 8, Sri Lanka; 2 No affiliation (Former Professor in Community Medicine, Faculty of Medicine, University of Colombo, Sri Lanka), Colombo, Sri Lanka

**Keywords:** Sri Lanka, Fertility goals, Unsafe abortion risk, Contraception, Case-control

## Abstract

**Background:**

When faced with an unintended pregnancy, some women choose to undergo an unsafe abortion, while others do not. This choice may depend on long-term contraception that shapes the fertility goals of women, along with many other risk factors. We assessed the risk for unsafe abortion associated with contraceptive practices based on women’s long-term behaviour, and its likely modification by the use of different types of contraceptives among women in Sri Lanka.

**Methods:**

An unmatched case-control study was conducted in nine hospitals among 171 women admitted for care following an unsafe abortion (Cases) and 600 women admitted to same hospitals for delivery of an unintended term pregnancy (Controls). Interviewer-administered-questionnaires assessed their socio-economic, reproductive and fertility (decisions on family size, family completion) characteristics, contraceptive method last used (traditional, modern), reasons for discontinuation/never-use, and contraceptive practices assessed at different time points.

Using several regression models, the risk of abortion was assessed for ‘non-use’ of contraception against ‘ineffective use’ at conception; for non-use further categorised as ‘never-use’, ‘early-discontinuation’ (discontinued before last birth interval) and ‘late-discontinuation’ (discontinued during last birth interval); and for any interaction between the contraceptive practice and contraceptive method last used among the ever-users of contraception.

**Results:**

At conception, ‘non-use’ of contraception imparted a two-fold risk for abortion against ineffective use (adjusted-OR = 2.0; 95% CI: 1.2–3.2). The abortion risk on ‘non-use’ varied further according to ‘early’ (adjusted-OR = 1.7; 95% CI: 1.1–3.1) and ‘late’ (adjusted-OR = 2.3; 95% CI: 1.5–3.6) discontinuation of contraception, but not with ‘never-use’ (crude-OR = 1.1; 95% CI: 0.6–2.3).

Among the ever-users, the risk of abortion varied within each contraceptive practice by their last used contraceptive method and reasons for discontinuation. A significant interaction between modern contraceptives and early discontinuation (adjusted-OR = 1.4; 95% CI = 1.1–3.1) demonstrated a seven-fold abortion risk for early discontinuation of modern methods against its ineffective use. In particular, hormonal methods seemed to be responsible for this risk (51.1% cases versus 42.5% controls).

**Conclusions:**

Long-term contraceptive practices showed varying risk for abortion, and was further modified by early discontinuation of modern contraceptives. This knowledge should be applied during postnatal visits by public-health staff.

**Electronic supplementary material:**

The online version of this article (doi:10.1186/s12884-017-1376-7) contains supplementary material, which is available to authorized users.

## Background

Over the past few decades, many low and middle income (LMI) countries have witnessed a demographic transition, brought about by the reduction of both birth and death rates. In South Asia, Sri Lanka reports a relatively low crude birth rate of 17.9 per 1000 population [1]. This is mainly due to the lowering of its total fertility rate from 5.3 in 1953 to 1.9 by 2000 [2, 3]. One of the main factors responsible for this fertility decline has been the reduction in marital fertility through integration of family planning services to the national maternal and child health programme [4, 5]. It has improved access to contraception, as evident by a rapid increase in the contraceptive use among married women aged 15–49 years from 32% in 1975 to 68% by 2006–7 [2, 6].

Despite the high prevalence of contraception, induced abortion has paradoxically persisted as an alternative fertility control method in Sri Lanka, accounting for a rate as high as 45 per 1000 women in the reproductive age (95% CI: 38–52/1000) [7–9]. Abortion is illegal in Sri Lanka, unless performed as a measure to save a pregnant woman’s life. As such, many of the abortions that take place are unsafe, carried out under septic conditions by unskilled providers, and lead to life-threatening complications [10, 11]. This calls for action to reduce the burden of unsafe abortions in Sri Lanka by identifying the unaddressed contraceptive needs of such women, which could serve as a cost-effective strategy for controlling fertility within the prevailing legal framework and socio-economic status of the country.

The unaddressed contraceptive needs of a woman who is not anticipating pregnancy include ‘non-use’ and ‘ineffective use’ of contraceptive methods [12]. Women resorting to either practice could equally contribute to an unintended pregnancy. However, not all but only the women having greater desire for achieving the intended spacing and family size would end-up with abortion, compared to others who choose to carry their unintended pregnancies to term. A comparison between these two groups of women on their contraceptive practices will reveal the exact unaddressed contraceptive need associated with abortion. For example, if higher non-use of contraception is observed among those who abort, it is an indication of the difficulties experienced during initiation or while being on methods available, whereas if higher ineffective use is observed, it could be due to poor quality or incorrect use of the contraceptive methods. Distinguishing which contraceptive practice would actually carry the risk for abortion is important, as the public health approaches used to address the two practices are rather different from each other. It would help us prioritise, to address women’s needs via targeted interventions, especially in low-resource settings. Many studies have explored the contraceptive practices of women who resort to abortion in order to identify the deficiencies in family planning programmes [11, 13–17]. However, since the practices of these women have not been compared against those of women continuing with an unintended pregnancy, most studies have failed to assess the exact role played by each contraceptive practice (‘non-use’ versus ‘ineffective use’) in the risk of abortion.

Furthermore, women not on contraception at the time of conception (‘non-users’) appear to be homogenous, yet they represent three different groups of women according to their behaviour prior to conception: women who have never used contraception (‘never-users’), those who have discontinued contraception before the last birth interval (i.e. no contraception used during last birth interval) (‘early discontinuers’) and those who have discontinued contraception during the last birth interval before the cycle of conception (‘late discontinuers’). Given these differences, the risk of abortion is further likely to vary with contraceptive practices differing according to the long-term behaviour of women. Testing this hypothesis would be beneficial for policy makers to identify the most vulnerable for unsafe abortion, amongst all women with no intention of getting pregnant.

It is shown that contraceptives that women discontinue early in life may be distinctly different from those that women discontinue at a later time [18]. As such, the risk of abortion associated with contraceptive practices is likely to be further modified by the type of contraceptive methods last used (traditional and modern methods). This hypothesis on risk modification among the ever users of contraception has not been explored in previous research, thus a preliminary assessment has the potential to formulate new knowledge on the modification of the risk of abortion associated with contraception.

A woman strives to achieve her primary fertility goal of desired spacing and family size via contraception [19]. Closely linked with this goal are several other risk factors identified for abortion in LMI countries, such as poor socio-economic status, marital status, achievements in reproduction (e.g. age at marriage and first child), ability to make decisions with partners on family size and family completion [11, 20–22]. As such, the risk of contraceptive practices for abortion as well as its effect modification ought to be assessed, independent of these risk factors that act as confounders.

This study aimed to identify the risk for unsafe abortion associated with contraceptive practices based on the long-term behaviour of Sri Lankan women, and the likely modification of this risk by the type of contraceptive last used among the ever users of contraception. Results are assumed to provide valuable evidence to similar countries with high illegal abortion rates.

## Methods

An unmatched case-control study was conducted in nine hospitals in eight out of 25 districts of Sri Lanka over a period of six months. Five of these hospitals were selected for the study based on the highest frequency of all types of abortions reported in the Indoor Morbidity and Mortality Registers for each district [Medical Statistics Unit: Indoor Morbidity & Mortality Registers, unpublished]. Two hospitals were purposively selected to ensure adequate representation of the minority Muslim and estate sector Tamil populations. In Colombo district, both apex referral tertiary hospitals located in the commercial capital were included.

‘Cases’ were women admitted to the selected hospitals following an unsafe abortion. The potential cases were identified by consecutive screening of women admitted to the gynaecology and medical/surgical casualty wards with signs and symptoms suggestive of an abortion. Of them, women with a confirmed diagnosis of ‘induced abortion’ were identified based on the World Health Organization (WHO) criteria [23] and triangulated during in-depth interviews with each woman, under three categories: ‘certainly’, ‘probably’ and ‘possibly’ induced abortions. Details on the definition and sampling used to identify unsafe abortions are published elsewhere [24].

The ‘unmatched controls’ were postpartum mothers admitted to postnatal wards following the delivery of a term unintended pregnancy, which was defined by the pregnancy of a woman contracepting during the cycle of conception or not contracepting due to reasons other than desired pregnancy [23]. They were selected from the same hospitals using a systematic random sampling method (every fifth mother in the postnatal ward admission registers) during the same study period.

The study aimed at recruiting a minimum sample of 159 cases and 600 controls, based on 80% power to detect potential associations between cases and controls at 5% alpha error; 20% minimum probability of exposure in the controls; with an odds ratio (OR) of 2.0; and 1:4 unmatched case-control ratio.

Using an interviewer-administered-questionnaire (Additional file 1), data were collected from the participants at an exit interview. The interviews were specifically conducted for this study as a separate research arm of a study that addressed several research questions on abortion. Pre-intern medical graduates who were not involved in providing care in the ward collected the data, after being trained in recruiting and collecting data from women by a group of psychologists and experts in qualitative research.

Data obtained from all cases and controls included: socio-economic status (age, marital status, education, employment), reproductive characteristics (gravidity, number of living male and female children), fertility behaviour (decisions made with partners on spacing and family size, family completion), reasons for never-use or discontinuation of contraception, and their contraception behaviour assessed in relation to three time points: ever, during last birth interval and during the cycle of conception. Among the ever-users of contraception, method that had been last used was also assessed. Modern methods were products or medical procedures that interfere with reproduction from acts of sexual intercourse, while traditional methods were natural/biological methods of interference.

Women were categorised by their contraceptive practices as ‘Ineffective users’ and ‘Non-users’ of contraception. ‘Non-users’ were further categorised as ‘Never-users’ (women who had never used contraception), ‘Early discontinuers’ (women who had discontinued contraception before the last birth interval) and ‘Late discontinuers’ (women who had discontinued contraception during the last birth interval before the cycle of conception).

Ethics clearance was obtained from the Faculty of Medicine, University of Colombo. This ethics approval was submitted to the provincial directors of health services and directors of each of the participating hospitals, upon which permission and ethics approval were granted to conduct the study. Informed verbal consent was obtained from each participant. Since induced abortion is illegal in Sri Lanka, obtaining written consent would dissuade patients from participating in the study, and therefore informed verbal consent was chosen over written consent. Participation was voluntary and participants could withdraw at any time.

### Data analysis

Data were analysed using SPSS (Statistical Package for Social Sciences) version 20.

To assess the risk of abortion associated with individual factors (contraceptive practices, methods used, socio-economic and reproductive characteristics, fertility behaviour), all cases and controls were compared using crude odds ratio (OR) and 95% confidence interval (CI) in univariate analysis.

Next, to assess the risk of abortion associated with contraceptive practices after controlling for confounders, logistic regression analysis was performed to obtain adjusted OR. In the regression model (Model 1), case-control status was included as the dependent variable; and the main predictor (contraceptive practices categorised as ‘non-use’ and ‘ineffective use’) and confounders (all the factors significant in univariate analysis) as independent variables. The model was tested using backward likelihood ratio method at 0.05 probability of entry and 0.1 exit. Thereafter, the regression analysis was repeated (Model 2), with contraceptive practices further categorised as ‘never-use’, ‘early discontinuation’, ‘late discontinuation’ and ‘ineffective use’ to assess how the abortion risk would vary according to the long-term behaviour of cases and controls.

Finally, to assess how the type of contraceptive last used (traditional and modern methods) would modify the abortion risk associated with each contraceptive practice, three more regression models were developed among the ever-users of contraception: first with only the main predictor (type of contraceptive) and confounders as independent variables (Model 3), second with the addition of contraceptive practices (‘early discontinuation’, ‘late discontinuation’ and ‘ineffective use’) (Model 4), and third with the addition of an interaction term between contraceptive practice and type of contraceptive (Model 5). ‘Traditional method last used’ and ‘ineffective contraceptive practice’ were taken as the reference categories. If an interaction became significant in the model, to determine its joint effect with the respective contraceptive practice, odds ratio relative to the effect within the reference categories was calculated, by obtaining the exponential value (EXP) of the two beta coefficients of the contraceptive practice and its interaction.

Cases and controls were compared by the type of contraceptive last used among the ever users of contraception and reasons given for never-use/discontinuation among those who discontinued and never-users. Chi-square and Fisher tests were applied when assessing significance.

## Results

The study recruited 600 controls and 822 potential cases of ‘certainly’ induced (*n* = 122), ‘probably’ induced (*n* = 161) and ‘possibly’ induced (*n* = 539) abortion. Of them, all ‘certainly’ induced abortion cases (*n* = 122) and only the ‘probably’ induced abortion cases showing nearly definitive clinical signs of infection that required intravenous antibiotics (*n* = 49) were recruited as ‘cases’ (*n* = 171) for the study. This re-grouping ensured that they were all definite cases of unsafe abortion.

Mean age of the cases and controls was 30.6 years (SD = 6.3) ranging from 15 to 46 years. The majority of women were of Sinhalese ethnicity (67.1%), married (94.6%), poorly educated (58.2%) and unemployed (71.7%). Most (34%) were in their third pregnancy.

Contraceptive practices of cases and controls at different time points in their lives are shown in Table 1. Over 90% of the women had initiated contraception, with no significant difference seen between the two groups (93.6% cases versus 91.5% controls; *p* = 0.38). Thereafter, contraceptive use declined.

**Table 1:**
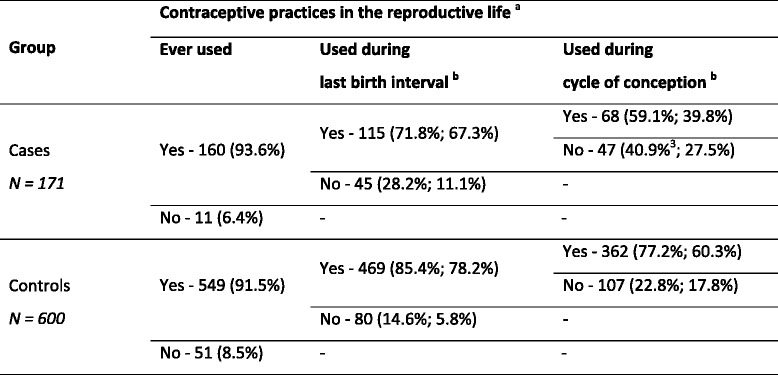
Comparison of the contraceptive practices between cases and controls at different time points in the reproductive life

^a^In parenthesis: (% out of the total in each category in the group; % out of the total in the group)

^b^In primigravid women, time since marriage was considered as their last birth interval

Table 2 shows the significant associations of cases and controls for unsafe abortion based on the univariate and logistic regression analyses. With regard to contraception, not being on contraceptives at the time of conception showed a two-fold risk for abortion against ineffective use (crude OR = 2.3; 95% CI: 1.6–3.3), which remained significant even after adjusting for potential confounders in the regression model (Model 1) (adjusted OR = 2.0; 95% CI: 1.2–3.2).

**Table 2:** Significant associations of all cases and controls for unsafe abortion

Risk factor	Cases *N* = 171		Controls *N* = 600		Crude OR (95% CI)	Adjusted OR (95% CI) Model 1^a,b^	Adjusted OR (95% CI) Model 2^a,c^
	No.	%	No.	%			
Contraceptive practice							
*At conception:*							
Non-use	103	60.2%	238	39.7%	2.3 (1.6–3.3)	2.0 (1.2–3.2)	
Ineffective use (Reference)	68	39.8%	362	60.3%	1.00	1.00	-
*According to long-term behaviour:*							
Never use	11	6.4%	51	8.5%	1.1 (0.6–2.3)		1.4 (0.7–3.1)
Early discontinuation	45	26.3%	80	13.3%	3.0 (1.9–4.7)		1.7 (1.1–3.1)
Late discontinuation	47	27.5%	107	17.8%	2.3 (1.5–3.6)		2.3 (1.5–3.6)
Ineffective use (Reference)	68	39.8%	362	60.3%	1.00	-	1.00
Secondary education							
Not completed	115	67.6%	332	55.5%	1.7 (1.2–2.4)	1.6 (1.1–2.4)	1.6 (1.1–2.4)
Completed (Reference)	55	32.4%	266	44.5%	1.00	1.00	1.00
Currently employed							
Yes	69	40.4%	149	24.8%	2.1 (1.4–2.9)	2.0 (1.3–2.9)	2.0 (1.3–2.9)
No (Reference)	102	59.6%	451	75.2%	1.00	1.00	1.00
Current marital status							
Single/divorce/separate/widow	31	18.1%	10	1.7%	12.9 (6.3–27)	9.4 (4.0–22)	9.7 (4.1–22.9)
Married (Reference)	140	81.9%	589	98.3%	1.00	1.00	1.00
Fertility behaviour							
No informed decision on family size	89	52.0%	173	28.8%	2.5 (1.6–3.8)	2.0 (1.2–3.2)	2.3 (1.5–3.4)
Informed decision made:							
Family completed	41	24.0%	230	38.3%	0.9 (0.5–1.4)	1.0 (0.6–1.6)	0.9 (0.5–1.4)
Family not completed (Reference)	41	24.0%	197	32.8%	1.00	1.00	1.00
Gravid status^d^							
Primigravid	36	21.1%	83	13.8%	1.7 (1.1–2.6)	2.5 (1.3–4.7)	2.8 (1.4–5.6)
Non-primigravid (Reference)	135	78.9%	517	86.2%	1.00	1.00	1.00
At least one female child present							
No	90	52.6%	210	35.0%	2.1 (1.5–2.9)	2.1 (1.4–3.2)	2.1 (1.4–3.3)
Yes (Reference)	81	47.4%	390	65.0%	1.00	1.00	1.00

^a^Logistic Regression (LR) model showing adjusted odds ratio (OR) of factors significant for unsafe abortion

^b^LR Model 1 with education level, employment, marital status, gravid status, fertility behaviour, having a female child and contraceptive practices in 2 categories: Non-use and Ineffective use as the independent variables; cases (unsafe abortion) and controls (term pregnancy) as the dependent variable

^c^LR Model 2 with all the variables of LR Model 1 except contraceptive practices in 4 categories: Early discontinuation, Late discontinuation, Never-use and Ineffective use

^d^Primigravid = Gravid women who have ever had one pregnancy; Non primigravid = Gravid women who have had more than one pregnancy

In further analysis of the contraceptive practices, the risk of abortion was three-fold with early discontinuation of contraceptives (crude OR = 3.0; 95% CI: 1.9–4.7) and two-fold with late discontinuation (crude OR = 2.3; 95% CI: 1.5–3.6), but none with never-use of contraceptives (crude OR = 1.1; 95% CI: 0.6–2.3). When adjusted for potential confounders in the regression model (Model 2), the risk of abortion remained further significant with both early discontinuation (adjusted OR = 1.7; 95% CI: 1.1–3.1) and late discontinuation (adjusted OR = 2.3; 95% CI: 1.5–3.6).

Among the ever-users, type of contraceptive last used was significant as a risk factor for abortion in univariate analysis (crude OR = 1.6; 95% CI: 1.1–2.3) but not in the regression analyses (Models 3–5) (Table 3). However, a significant interaction between the early discontinuation of contraception and the use of modern contraceptives (adjusted OR = 1.4; 95% CI = 1.1–3.1) was noted in Model 5. As a result, the joint risk of abortion associated with early discontinuation of modern contraceptives appeared to be almost seven-fold against its ineffective use [EXP (beta-coefficients of early discontinuation (1.6) + interaction (0.34)); calculation not shown in Table 3]. This risk was higher than the five-fold abortion risk seen with early discontinuation of traditional methods (adjusted OR = 4.9; 95% CI: 1.7–14.4). No such interaction was observed with late discontinuation.

**Table 3:** Significant associations of cases and controls among ever-users of contraception for unsafe abortion

Risk factor	Cases *N* = 160		Controls *N* = 549		Crude OR (95% CI)	Adjusted OR (95% CI) Model 3^a^	Adjusted OR (95% CI) Model 4^b^	Adjusted OR (95% CI) Model 5^c^
	No.	%	No.	%				
Long-term contraceptive practices								
Early discontinuation	45	26.3%	80	13.3%	3.0 (1.9–4.7)		1.75 (1.1–3.2)	4.9 (1.7–14.4)
Late discontinuation	47	27.5%	107	17.8%	2.3 (1.5–3.6)		2.3 (1.4–3.6)	3.5 (1.6–7.6)
Ineffective use (Reference)	68	39.8%	362	60.3%	1.00	-	1.00	1.00
Type of contraceptive last used								
Modern method	117	73.1%	349	63.6%	1.6 (1.1–2.3)			
Traditional method (Reference)	43	26.9%	200	36.4%	1.00	NS^e^	NS^e^	NS^e^
Interaction (Contraceptive practice & Type of contraceptive (*n* = 378)								
Early discontinuation & modern method	37	39.8%	60	21.0%				1.4 (1.1–3.1)
Late discontinuation & modern method	34	36.6%	76	26.7%				1.7 (0.8–5.0)
Ineffective use & traditional method (Reference)	22	23.6%	149	52.3%	-	-	-	1.00
Secondary education								
Not completed	105	67.6%	300	54.7%	1.6 (1.1–2.3)	1.6 (1.1–2.5)	1.6 (1.1–2.4)	1.7 (1.1–2.5)
Completed (Reference)	55	32.4%	249	45.3%	1.00	1.00	1.00	1.00
Currently employed								
Yes	64	40.0%	140	25.5%	1.9 (1.3–2.8)	1.7 (1.1–2.5)	1.8 (1.2–2.7)	1.8 (1.2–2.7)
No (Reference)	96	60.0%	409	74.5%	1.00	1.00	1.00	1.00
Current marital status								
Single/divorce/separate/widow	29	18.1%	10	1.8%	11.9 (5.7–25)	9.4 (4–21.9)	9.3 (3.9–22.4)	9.9 (4.1–24.3)
Married (Reference)	131	81.9%	539	98.2%	1.00	1.00	1.00	1.00
Fertility behaviour								
No informed decision on family size	79	49.4%	145	26.4%	2.3 (1.5–3.7)	2.1 (1.3–3.4)	1.9 (1.2–3.2)	1.9 (1.1–3.1)
Informed decision made:								
Family completed	40	25.0%	224	40.8%	0.8 (0.5–1.3)	0.8 (0.5–1.4)	0.9 (0.5–1.4)	0.8 (0.5–1.4)
Family not completed (Reference)	41	25.6%	180	32.8%	1.00	1.00	1.00	1.00
Gravid status^d^								
Primigravid	36	22.5%	83	15.1%	1.6 (1.1–2.5)	2.5 (1.3–4.8)	2.8 (1.1–5.6)	2.1 (1.0–4.4)
Non-primigravid (Reference)	124	77.5%	466	84.9%	1.00	1.00	1.00	1.00
At least one female child present								
No	84	52.5%	194	35.3%	2.0 (1.4–2.9)	2.2 (1.4–3.4)	2.1 (1.3–3.3)	2.1 (1.3–3.3)
Yes (Reference)	76	47.5%	355	64.7%	1.00	1.00	1.00	1.00

^a^LR Model 3 showing adjusted odds ratio (OR) of the factors significant for unsafe abortion among ever-users of contraception: education, employment, marital status, gravid status, fertility behaviour, female child and type of contraceptive last used as the independent variables; cases (unsafe abortion) and controls (unintended term pregnancy) as the dependent variable

^b^LR Model 4 (all variables of LR Model 3 plus contraceptive practices)

^c^LR Model 5 (all variables of LR Model 4 plus an interaction term between contraceptive practices and contraceptive method)

^d^Primigravid = Gravid women who have ever had one pregnancy; Non primigravid = Gravid women who have had more than one pregnancy

^e^NS= Variable was not significant in the regression model

**Fig. 1:**
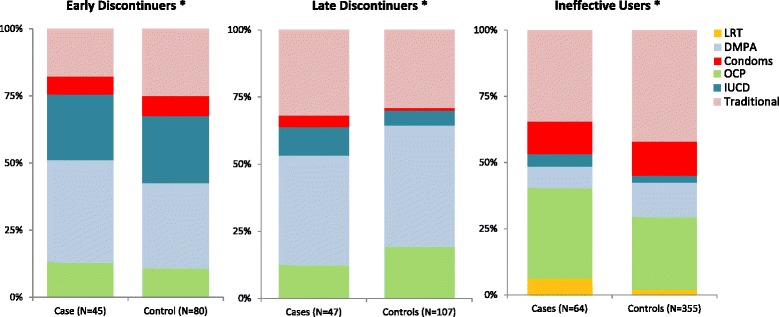
Comparison of the cases and controls by the contraceptive types last used among ever-users of contraception. *Chi-square (yates corrected) test applied to compare cases and controls by three types of contraceptives last used: traditional, hormonal and non-hormonal within each group of women of early discontinuation, late discontinuation and ineffective use of contraception

Among the ever-users of contraception (Fig. 1), compared to controls, a higher proportion of cases had last used modern hormonal methods (51.1% cases versus 42.5% controls, *p* = 0.35) such as depot medroxy-progesterone preparations (DMPA) and oral contraceptive pills (OCP) among the early discontinuers, in contrast to modern non-hormonal methods (17.5% cases versus 6.5% controls, *p* = 0.09) such as intra-uterine-devices (IUCD) and condoms among the late discontinuers. A higher proportion of cases among the ineffective users failed on modern methods (65.6% cases versus 58.0% controls) (*p* = 0.33). None of the differences were significant.

Reasons given for discontinuing or never using contraceptives varied among cases and controls (Table 4). Among all those who discontinued, pregnancy intention was higher among cases compared to controls (OR = 11.9; 95% CI: 2.6–55.2; *p* < 0.01). Though inadequate knowledge on contraceptive methods (*p* = 0.55) and perception on using a method being ‘inconvenient’ (*p* = 0.57) were higher among cases, these were not significant among the never-users.

**Table 4:** Comparison of cases and controls in the never users and discontinuers by their reasons for discontinuation or never use of contraception

Characteristics	Never-users^a^		Discontinuers^a^	
	Cases *N* = 11	Controls *N* = 51	Cases *N* = 92	Controls *N* = 187
Inadequate knowledge:	36.4%	27.4%	NA	NA
Opposition to use (self/partner):	13.5%	21.9%	NA	NA
Religious prohibitions:	0.0%	4.2%	NA	NA
Fertility related reasons:				
• Wanted to become pregnant	2.7%	8.3%	**11.9%**	**1.1%**
• Infrequent sex/partner away	10.8%	6.2%	8.7%	4.8%
• Fear of becoming sub-fertile	0.0%	0.0%	2.2%	2.7%
• Assumed to be infertile	2.7%	1.0%	4.3%	8.6%
Method related reasons:				
• Medical contraindications	9.1%	15.7%	3.3%	8.6%
• Side effects of contraception	0.0%	0.0%	**14.1%**	**25.1%**
• Access/availability of service	2.7%	2.1%	5.4%	3.7%
• Perceived as inconvenient to use	8.1%	1.0%	3.3%	2.7%
• Cost too much	0.0%	0.0%	3.3%	0.5%
• Other (breastfeeding, widow)	0.0%	2.1%	7.6%	8.6%
No reason given:	0.0%	0.0%	31.5%	27.8%

^a^All values given as percentages; Chi-square (Yates corrected) and Fisher tests applied for significance of associations; significance at *p* < 0.05 given in bold letters; NA=not applicable

## Discussion

Our study showed that discontinuation of contraception was associated with greater risk of unsafe abortion compared to ineffective use among Sri Lankan women, after adjusting for potential confounders. Never–use of contraception did not impart any risk for abortion. Modern contraceptives seemed to modify the relationship of abortion with early discontinuation. Overall, the study confirmed that women discontinuing contraception were more motivated than never users and ineffective users to prevent a pregnancy, so that they continued with their desired fertility goal through abortion.

Ineffective use of contraception has been commonly identified among abortion seekers in Sri Lanka [8, 11] and elsewhere [25]. In contrast, our study identified a two-fold risk for unsafe abortion among women not using any contraceptive method at the time of conception, against women who used a method ineffectively. This may imply that the actual use in terms of women years protected may not be as high as the contraceptive prevalence reported for Sri Lanka. It further implies the difficulties faced by women in either initiating contraception or being on/obtaining a contraceptive method long-term. On the other hand, our findings being less in favour of any abortion risk associated with inefficient use of contraceptive methods [26] provides fewer implications on the programmatic issues related to the quality of contraceptive methods in terms of safety, efficiency and ease of use.

We have further shown that the risk of abortion associated with non-use was specifically high among women who had discontinued contraceptives, but not among those who had never used. This reflects the poor follow-up of women already on contraceptives who may experience difficulties while being on a method. The Government of Sri Lanka runs a free healthcare service, through which family planning services are provided free of charge at every primary healthcare facility in the country. At grass root level, the Public Health Midwife (PHM) provides domiciliary and clinic care on family planning via a well-established public health network. Despite such wide access to family planning services, statistics show that nearly 1/3rd of contraceptive users in Sri Lanka discontinue a method within 12 months of adoption [2]. To improve this situation, PHMs’ domiciliary care should be strengthened to target not only the new-acceptors of contraception but also the ones who subsequently discontinue. PHMs should be further trained on counselling skills, which are proven to be effective for improving compliance [27].

Our study highlights the risk of abortion being almost similar with late discontinuation (adjusted OR = 2.3) and early discontinuation (adjusted OR = 1.7). The risk associated with early discontinuation suggests that the window-period most optimal for reinforcing contraception by PHM would be the postpartum period, during which she should review the difficulties that women had previously experienced with contraceptives and offer alternatives accordingly. Women would be easily accessible to PHM during this period as they are expected to regularly visit poly clinics that address the needs of both mother and baby. Those who discontinued early being at such risk of abortion highlights these missed opportunities in the prevention of unsafe abortions among Sri Lankan women.

In Sri Lanka, 53% of 15–49 year old married women rely on modern methods and 16% on traditional methods [2]. Interestingly, our study sheds light on modern contraceptives further modifying the risk of abortion associated with early discontinuation (the abortion risk increasing from five-fold (adjusted OR = 4.9) to seven-fold (adjusted OR = 6.9), if the discontinued contraceptives were modern methods. Further according to our study, this risk was seen to be higher predominantly for discontinuation of hormonal methods such as injectables and pills. In concurrence, studies on abortion seekers have consistently shown that modern contraceptives especially injections and pills are the most frequently discontinued methods [25] and most often due to side effects [2]. Features of contraceptives preferred by women are effectiveness and lack of side effects, which will ensure increased compliance [28], thus expanding effective contraceptive choices is recommended for preventing unsafe abortions [7, 29]. In this regard, IUCD plays an important role as it shows high continuation and satisfaction, despite the initial general dislike for invasive methods or fear of uterine infection/rupture [15]. As such, women should be encouraged not merely to choose any modern contraceptive method but to specifically select a long acting one such as IUCD to prevent many of the discontinuations.

As much as the timely reinforcement of contraception after child birth, following them up thereafter also seem to play an important role in late discontinuation. A PHM’s family planning services include providing awareness, counselling and methods to clients using a cafeteria approach, through which contraception already initiated could be further sustained. However, in the current health care system, once initiated, regular monitoring of women’s contraceptive needs is weak, thus providing fewer opportunities for addressing the difficulties faced by women with regards to maintenance of contraception. Among those who discontinued late, the abortion risk was significantly higher for non-hormonal methods such as condoms and IUCD (Fig. 1). Although such methods did not modify the abortion risk associated with late discontinuation, the findings may help to formulate hypothesis on differential contraceptive preferences within each contraceptive practice leading to abortion, so that challenges faced by women could be better framed.

### Study strengths and limitations

This study obtained highly reliable data collected by unbiased data collectors not involved in providing care. Misclassification for cases was minimised by using a tested definition for unsafe abortions, while the controls were appropriately selected from the same hospitals as cases in order to minimise selection bias and potential confounders. Validated data collection tools enhanced the quality of data. However, this study was limited to women presenting to hospital following unsafe abortions. Thus, the findings may not apply to women who had safely induced abortions in Sri Lanka.

Owing to smaller size, statistical power was not adequate for a detailed analysis of the abortion risk on individual methods used by ever-users resorting to each contraceptive practice and the reasons for never-use given by never-users, thus requires cautious interpretation. However, we believe that this study was able to formulate several hypotheses on these aspects and shed light for testing them in a larger study designed with adequate power.

## Conclusions

Non-use of contraceptive methods at the time of conception showed a two-fold risk for unsafe abortion against its ineffective use, highlighting the vulnerability of women to abortion due to difficulties faced in either initiation or continuation of long-term contraception, rather than due to poor quality or incorrect use of contraceptive methods. In further comparison, this risk was more apparent with ‘early’ as well as ‘late’ discontinuation of contraception, but not with never-use, highlighting the need for better follow-up of women on contraception. Modern contraceptives seemed to further increase the risk of unsafe abortion with early discontinuation.

Results point to the need for strengthened domiciliary care by health care workers, tailored to help women select an appropriate method according to their preference and constraints, while considering the postpartum period as a window for reinforcing contraception. Further research is recommended to explore the hypotheses formulated on differential contraceptive preferences in modifying the risk of abortion.

## Additional file

Additional file 1: Interviewer administered questionnaire. Data collection tool used to collect data from cases and controls. (PDF 124 kb)

## Abbreviations

WHO: World Health Organization
OR: Odds ratio
SD: Standard deviation
CI: 95% confidence interval
IUCD: Intra-uterine-devices
DMPA: Depot medroxy-progesterone preparations
OCP: Oral contraceptive pills
LRT: Ligation & resection of tubes
