# Supplementary material for: Risk of unsafe abortion associated with long-term contraception behaviour: a case control study from Sri Lanka
Source: BMC Pregnancy Childbirth. 2017 Jun 29;17:205. doi: 10.1186/s12884-017-1376-7 (PMC5493010; doi:10.1186/s12884-017-1376-7)
Supplement: Additional file 1: — Interviewer administered questionnaire. Data collection tool used to collect data from cases and controls. (PDF 124 kb) [file 12884_2017_1376_MOESM1_ESM.pdf]

SERIAL NO: . . . .   TYPE: CASE / CONTROL

STUDY QUESTIONNAIRE

SECTION I: SOCIO-DEMOGRAPHIC CHARACTERISTICS

1.1 Identification No. ☐☐

1.2 What is the age that you completed at your last birthday? ☐☐ n complete years

1.3 What ethnic group do you belong to?

Sinhala – 1 | Tamil – 2
Muslim – 3 | Other – 4 (specify: ……………………….)

1.4 What religion do you belong to?

Buddhist – 1 | Hindu – 2
Catholic/ Christian – 3 | Islam – 4
Other – 5 (specify: ……………………….)

1.5 What is your current marital status?

Married (customary or legal) – 1 | Single – 2
Widowed – 3 | Divorced/ separated – 4

1.6 Have you ever attended school? Yes – 1 | No – 2 (skip to Q.1.8)

1.7 What is your highest level of education?

Grade 1-5 – 1 | Grade 6-10 – 2
Passed O/Levels – 3 | Grade 11-13 – 4
Passed A/Levels – 5 | Technical/Diploma/vocational training – 6
University Degree – 7 | No answer – 8

1.8 What is your current status of employment?

Currently employed (also includes self employment) – 1
Previously employed – 2 (skip to Q.2.1)
Never employed – 3 (skip to Q.2.1)

1.9 What kind of work do you mainly do in your present occupation? Specify the work.

…………………………………………………………………………...

SECTION II: FAMILY FORMATION PATTERNS

The following questions are about your family formation pattern.

2.1 At what age did you first get married (customary or legal)? ☐☐ Years old

2.2 Please give details of your pregnancies.

Pregnancy	Age at pregnancy (in years)	Outcome of pregnancy *	If living child	
			Age	Sex (Boy/Girl)
a. First				
b. Second				
c. Third				
d. Fourth				
e. Fifth				
f. Sixth				

* LB (live birth), SB (still birth), SA (spontaneous abortion), IA (induced abortion), EP (ectopic pregnancy), MP (molar pregnancy)

2.3 Have you ever discussed with your husband/partner about the number of children you desire to have in the family?

Yes – 1   No – 2 (skip to Q. 3.1)

2.4 Have you decided with your husband/partner about the number of children you desire to have in the family?

Yes – 1 (specify the no: ……)   No decision made yet – 2 (skip to Q.3.1)

2.5 Have you completed your desired family size?

Yes – 1   Not yet – 2

SECTION III: CONTRACEPTIVE HISTORY

The following questions are about your contraceptive history.

3.1 Women are using various methods to plan their pregnancies. Have you ever used any contraceptive method/methods to delay or avoid pregnancy?

Yes –1   No – 2 (skip to Q.3.7)

3.2 [Obtain information on whether she was using any type of contraceptive method at the following time points: before marriage, before first pregnancy and during intervals between the subsequent pregnancies, up to the time of her last pregnancy, which ended up as an abortion or continued as an unplanned pregnancy].

………………………………………………………………………………………………
………………………………………………………………………………………………
………………………………………………………………………………………………

3.3 What was the contraceptive method that you have **last used**?

....

3.4 Were you using this contraceptive method during the last birth interval (time interval between your last pregnancy which has currently ended as an abortion or continued as an unintended pregnancy, and the pregnancy immediately before that)?

Yes – 1   No – 2 (skip to Q.3.7)

No previous pregnancy – 3 (skip to Q. 3.7)

3.5 Were you using this contraceptive method during the month of conception of your last pregnancy (that is around the time when you last became pregnant, which has currently ended as an abortion or continued as an unintended pregnancy)?

Yes -1   No – 2 (skip to Q.3.7)

3.6 [Obtain information on likely reasons for failure of this method].

……………………………………………………………………………………………..
……………………………………………………………………………………………

3.7 [Obtain information on reasons for discontinuation of/ not ever using contraception].

………………………………………………………………………………………………
……………………………………………………………………………………………
………………………………………………………………………………………………
